# Alterations of Gut Microbiome and Serum Metabolome in Coronary Artery Disease Patients Complicated With Non-alcoholic Fatty Liver Disease Are Associated With Adverse Cardiovascular Outcomes

**DOI:** 10.3389/fcvm.2021.805812

**Published:** 2022-01-03

**Authors:** Xiaomin Hu, Ruilin Zhou, Hanyu Li, Xinyue Zhao, Yueshen Sun, Yue Fan, Shuyang Zhang

**Affiliations:** ^1^Department of Cardiology, Department of Medical Research Center, State Key Laboratory of Complex Severe and Rare Diseases, Peking Union Medical College Hospital, Chinese Academy of Medical Science and Peking Union Medical College, Beijing, China; ^2^Department of Cardiology, State Key Laboratory of Complex Severe and Rare Diseases, Peking Union Medical College Hospital, Chinese Academy of Medical Science and Peking Union Medical College, Beijing, China

**Keywords:** coronary heart disease, non-alcoholic fatty liver disease, microbiome, metabolome, cardiovascular outcomes

## Abstract

**Rationale:** Patients suffering from coronary artery disease (CAD) complicated with nonalcoholic fatty liver disease (NAFLD) present worse cardiovascular outcomes than CAD patients without NAFLD. The progression of CAD is recently reported to be associated with gut microbiota and microbe-derived metabolites. However, it remains unclear how the complication of NAFLD will affect gut microbiota and microbe-derived metabolites in CAD patients, and whether or not this interplay is related to the worse cardiovascular outcomes in CAD-NAFLD patients.

**Methods:** We performed 16S rRNA sequencing and serum metabolomic analysis in 27 CAD patients with NAFLD, 81 CAD patients without NAFLD, and 24 matched healthy volunteers. Predicted functional profiling was achieved using PICRUSt2. The occurrence of cardiovascular events was assessed by a follow-up study. The association of alterations in the gut microbiome and metabolome with adverse cardiovascular events and clinical indicators was revealed by Spearman correlation analysis.

**Results:** We discovered that the complication of NAFLD was associated with worse clinical outcomes in CAD patients and critical serum metabolome shifts. We identified 25 metabolite modules that were correlated with poor clinical outcome in CAD-NAFLD patients compared with non-NAFLD patients, represented by increased cardiac-toxic metabolites including prochloraz, brofaromine, aristolochic acid, triethanolamine, and reduced potentially beneficial metabolites including estradiol, chitotriose, palmitelaidic acid, and moxisylyte. In addition, the gut microbiome of individuals with CAD-NAFLD was changed and characterized by increased abundances of *Oscillibacter ruminantium* and *Dialister invisus*, and decreased abundances of *Fusicatenibacter saccharivorans, Bacteroides ovatus* and *Prevotella copri*. PICRUSt2 further confirmed an increase of potential pathogenic bacteria in CAD-NAFLD. Moreover, we found that variations of gut microbiota were critically correlated with changed circulating metabolites and clinical outcomes, which revealed that aberrant gut microbiota in CAD-NAFLD patients may sculpt a detrimental metabolome which results in adverse cardiovascular outcomes.

**Conclusions:** Our findings suggest that CAD patients complicated with NAFLD result in worse clinical outcomes possibly by modulating the features of the gut microbiota and circulating metabolites. We introduce “liver-gut microbiota-heart axis” as a possible mechanism underlying this interrelationship. Our study provides new insights on the contribution of gut microbiota heterogeneity to CAD-NAFLD progression and suggests novel strategies for disease therapy.

**Graphical Abstract:**
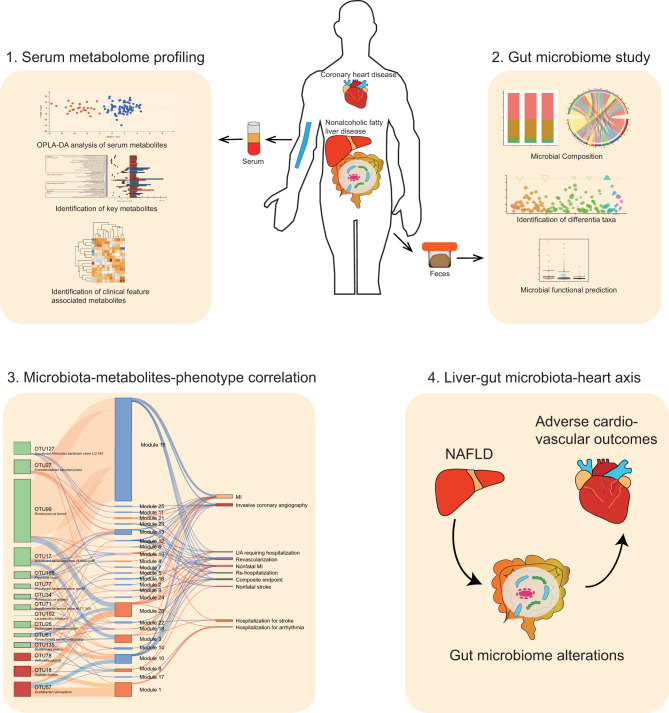


## Introduction

Non-alcoholic fatty liver disease (NAFLD) is a leading contributor to the growing burden of chronic liver disease globally (1, 2). NAFLD begins with the aberrant accumulation of triglycerides in the liver, which in some individuals elicits an inflammatory response that can progress to cirrhosis and liver cancer (3). Moreover, NAFLD causes considerable liver-related and extrahepatic morbidity and mortality worldwide (4, 5). The increasing prevalence of NAFLD has been associated with obesity (6), type 2 diabetes mellitus (T2DM) (7), hyperlipidemia, hypertension (HTN) (7), and elevated liver enzymes, namely, alanine aminotransferase (ALT) (6) and γ-glutamyl transferase (GGT) (8). The strong association of fatty liver disease with metabolic syndrome has stimulated interest in the putative role of fatty liver disease in the progression of cardiovascular disease. Accumulating clinical and epidemiological evidence indicates that NAFLD is associated with an increased risk of cardiovascular disease and that NAFLD dictates the outcome of patients with coronary artery disease (CAD) more frequently and to a greater extent than does the progression of CAD itself (9–11). NAFLD detected with ultrasonography is strongly associated with increased intimal medial thickness of the carotid artery and an increased prevalence of carotid atherosclerotic plaques (12–14). Moreover, CAD patients complicated with NAFLD had a significantly higher 10-year risk of cardiovascular events than those with CAD alone (15). Recently, NAFLD has been proposed to be an independent risk factor for CAD (16). However, to our knowledge, the nature and extent of the associations between NAFLD and adverse outcomes in CAD patients is not clear.

Recent work has begun to elucidate the potential contribution of NAFLD to CAD progression and outcome. Some evidence suggests that NAFLD might play a part in the pathogenesis of cardiovascular disease through the systemic release of several inflammatory (17), hemostatic, and oxidative stress mediators or by contributing to atherogenic dyslipidemia (11). Recent years, with the exploration of liver-gut microbiome axis (18), the potential role of gut microbiomes in liver disease was paid attention on. Recent studies showed that NAFLD and its severity were associated with a high abundance of inflammatory bacterial products (19). Species belonging to the family *Enterobacteriaceae* and the genera *Streptococcus* and *Gallibacterium* were enriched in NAFLD (17). One research showed significantly increased *Copococcus* and *Veillonella parvula* abundance and significantly decreased *Ruminococcus gnavus* and *Bacteroides dorei* abundance in CAD-NAFLD patients compared with CAD patients without NAFLD (20). These studies have provided evidence that gut microbiota might contribute to the progression of CAD-NAFLD. However, the relationship between gut microbiota, circulating metabolites and the prognosis of CAD-NAFLD patients remained ambiguous.

To address the questions above, we performed a multi-omics study on 108 CAD patients (CAD-NAFLD group *N* = 27 and CAD group *N* = 81) and 24 healthy controls. We conducted 16S rRNA sequencing to analyze the gut microbial characteristics, untargeted liquid chromatography-mass spectrometry (LC-MS) to analyze the serum metabolic profiles and a follow-up study to reveal the outcomes of the patients. The objectives of this study were to identify the specific features of the gut microbiota and host metabolite profiles in CAD-NAFLD patients, and reveal the interactions between gut microbiome, host circulating metabolites, and incidence of cardiovascular events.

## Methods

### Study Participants and Sample Collection

We consecutively recruited 24 healthy volunteers and 108 CAD patients who were hospitalized for coronary angiography at Peking Union Medical College Hospital (PUMCH). Patients who exhibited ≥ 50% stenosis in at least one main coronary artery were diagnosed with CAD. The 108 CAD patients were further split into the following two subgroups basing on whether complicated with NAFLD or not: (1) CAD (*N* = 81) and (2) CAD-NAFLD (*N* = 27). CAD-NAFLD patients are defined as CAD patients who were also diagnosed with NAFLD. For the diagnosis of CAD and NAFLD, please refer to our previous study (21) and Supplementary Materials, respectively. For controls, we enrolled subjects who were identified as having no CAD-related clinical signs or symptoms. Subjects were excluded if they had gastrointestinal diseases, malignant tumors, autoimmune disorders, infectious diseases, renal dysfunction (creatinine > 3.0 mg/dl), a history of gastrointestinal surgery in the previous year or were administered antibiotics for more than 3 days in the previous 3 months.

Peripheral venous blood was collected from the participants in the morning of the day after admission, and all clinical information was collected. Participants were given a stool sampler and provided detailed illustrated instructions for sample collection. Freshly collected stool samples from each participant were immediately transported to the laboratory and immediately frozen at−80°C. The detailed method for blood collection, stool collection and clinical information collection methods refer to our previous study (21). The study was performed in accordance with the principles of the Declaration of Helsinki. Subjects provided written, informed consent for participation in the study.

### Follow-Up Study

Patient follow-up was conducted when they were reviewed in the clinic or through telephone interviews with patients/their close family members. The composite endpoint of this study consisted of all-cause mortality and/or reoccurrence of myocardial infarction (MI) and/or stroke and/or readmission for major adverse cardiac events (MACEs). New hospitalization and/or all-cause mortality was identified with the electronic medical record system of PUMCH or interviews with the patient (or a family member) in cases of events outside of PUMCH. Binary logistic regression analysis was utilized to explore the relationship between the complications of NAFLD and the outcome of CAD patients after adjusting for potential confounding factors. Statistical analysis was performed using SPSS statistics software (v24.0, SPSS Inc., Chicago, IL, USA). The results of binary logistic regression were visualized as forest plots using the R package ggplot2.

### Untargeted Metabolomics Study

Sample analysis was performed by a Waters ACQUITY ultra-high-performance liquid chromatography system (Milford, MA, USA) coupled with a Waters Q-TOF Micromass system (Manchester, UK). Sample analysis was performed in both positive and negative ionization modes. The detailed procedures for sample preparation, LC-MS (Liquid chromatography-mass spectrometry) experiments, and peak-ion intensity matrix preparation were described in our previous publication (21). The matrix was further reduced by removing peaks with missing values in more than 80% of the samples and those with isotope ions from each group to obtain consistent variables. The coefficient of variation (CV) of metabolites in the quality control (QC) samples was set at a threshold of 30% for the assessment of repeatability in the metabolomics datasets. Then, we used the Wilcoxon rank-sum test to identify peaks that differed between the CAD and CAD-NAFLD groups. Next, we performed orthogonal partial least squares discriminant analysis (OPLS-DA) by SIMCA software (v14.1, Umetrics, Sweden). Significant metabolites were selected on the basis of variable importance in the projection (VIP) value > 1 and *P* < 0.05. Annotation was achieved by online databases, including the online HMDB database (http://www.hmdb.ca)(version:4.0) (22). MetaboAnalyst (http://www.metaboanalyst.ca) (version 4.0) was used for the identification of metabolic pathways (23). Although 132 patients were recruited, the metabolite information of three people in the HC group was not collected. Thus, 129 samples were used in the metabolome analysis.

### Extraction of DNA and Sequencing of the 16S RRNA V3-V4 Region

Bacterial DNA was isolated from fecal samples by using the bead-beating method. The DNA extracted from each sample was used as the template to amplify the V3-V4 region of 16S rRNA gene by using PCR (Phusion High-Fidelity PCR Master Mix with GC Buffer, from New England Biolabs Company), which were illustrated in our previous publication (24). Raw data quality control was performed. A sequencing library of the V3-V4 regions of the 16S rRNA gene was prepared. The purified products were mixed at an equal ratio for sequencing using an Illumina MiSeq system (Illumina Inc., USA). The detailed method refers to our previous study (24).

### Sequencing Data Analysis

Operational taxonomic units (OTUs) were clustered at the cutoff of 97% by using USEARCH v.8.0. The protocol can be found on the website (http://drive5.com/usearch/manual/uparse_pipeline.html).

The taxonomic composition of each group was visualized as a stacked bar plot at the phylum level and as a chord plot at the genus level with the ggplot2 package. For comparisons among groups, edgeR was utilized to identify significantly differential features, and the Benjamini-Hochberg method was used to control the FDR. Phylogenetic Investigation of Communities by Reconstruction of Unobserved States (PICRUSt2) was utilized to predict the metagenomic functional compositions. Pathways that were different in abundance between the CAD and the CAD-NAFLD groups were obtained using Welch's *t*-test by STAMP software (v2.1.3). The visualization of the identified pathways was obtained by using the pheatmap package. To obtain functional predictions based on the 16S rRNA sequences, the taxonomic classification of sequences based on the Greengenes database was performed using *usearch -otutab*. Then, Bugbase was used to predict the phenotypes of the bacterial community (25).

### Spearman Multiomic Correlation Analysis

Spearman correlations between important bacterial taxa, serum metabolites and clinical parameters were calculated by using SPSS 24.0. The correlations between features were visualized using the pheatmap package. Multiple omics correlations were visualized in a Sankey plot by utilizing the R package networkD3.

## Results

### Participant Characteristics

The characteristics and traditional cardiovascular risk factors of the participants are summarized in Table 1. In terms of disease severity, the difference in Gesini score [an indicator for atherosclerotic burden quantification (26)] was not significant, although the CAD-NAFLD group presented a much higher percentage of 3 stenosed vessels (51.85%) than the CAD group (26.91%). Moreover, biomarkers of myocardial injury and inflammation such as cardiac troponin I (cTnI) (27) and high-sensitivity C-reactive protein (hsCRP) (28) presented no significant difference between CAD-NAFLD group and CAD group. As for medication, CAD-NAFLD group has a significantly higher percentage of patients taking statins (55.56%) and oral antidiabetic drugs (OAD) (29.63%) than CAD group (48.15% for statins and 19.75% for OAD). In aspect of NAFLD-related clinical indexes such as triglyceride (TG), total cholesterol (TC), high density lipoprotein cholesterol (HDL-C), low density lipoprotein cholesterol (LDL-C), CAD-NAFLD group and CAD group showed no significant difference, this may due to the medication of CAD-NAFLD patients. Overall, the difference in disease severity baseline information showed was inconspicuous in the diseased groups and clinical indexes were not significantly affected by the complication of NAFLD at baseline.

**Table 1:** Characteristics of the study cohort.

	**Healthy control (*n* = 24)**	**CHD (*n* = 81)**	**CHD-NAFLD (*n* = 27)**	***P-*value**
Age, years^#^	54.75 (48.25, 59.5)	61.78 (52.5, 70)	62.8 (60, 68)	<0.001^a^^c^
Female^$^	7 (25.93)	20 (24.69)	15 (62.5)	<0.002^a^^c^
SBP, mmHg^*^	119.17 ± 8.61	131.30 ± 18.27	129.15 ± 14.19	<0.001^a^^c^
BMI, kg/m2^*^	23.94 ± 2.59	26.18 ± 3.39	26.48 ± 3.18	<0.004^a^^c^
Waistline, cm^*^	80.58 ± 8.69	92.80 ± 9.13	95.48 ± 8.17	<0.001^a^^c^
Current smoker^$^	0 (0)	28 (34.57)	9 (33.33)	<0.001^a^^c^
Drinking history^$^	1 (4.17)	43 (53.09)	16 (59.26)	<0.001^a^^c^
No. of stenosed vessels				<0.05^a^^b^^c^
NA	NA	2 (2.47)	2 (7.4)	
1	NA	18 (22.22)	7 (25.93)	
2	NA	23 (28.39)	4(14.81)	
3	NA	38 (26.91)	14 (51.85)	
Gesini score^#^	NA	44.67 (19, 63)	32.29 (15, 44)	
**Medication**
Statins^$^	0 (0)	24 (29.63)	15 (55.56)	<0.05^a^^b^^c^
Antihypertensive drugs^$^	4 (16.67)	54 (66.67)	15 (55.56)	<0.001^a^^c^
Oral antidiabetic drugs^$^	0 (0)	16 (19.75)	13 (48.15)	<0.05^a^^b^^c^
**Laboratory data**
TG, mmol/l^#^	1.49 (0.83, 1.87)	1.73 (1.04, 1.88)	1.73 (1.03, 2.58)	
TC, mmol/l^#^	4.78 (4.1, 5.43)	4.13 (3.22, 4.76)	3.97 (3.25, 4.36)	<0.001^c^
HDL-C, mmol/l^#^	1.22 (0.97, 1.37)	0.98 (0.81, 1.1)	0.93 (0.83, 1.08)	<0.01^a^^c^
LDL-C, mmol/l^#^	2.84 (2.45, 3.24)	2.40 (1.7, 2.75)	2.19 (1.64, 2.44)	<0.001^c^
FBG, mmol/l^#^	6.58 (6.63, 7.05)	7.28 (5.75, 8.2)	7.77 (6, 8.6)	<0.05^c^
CR, μmol/l^#^	67.625 (62, 75.25)	84.23 (71, 91)	74 (60, 86)	<0.01^a^
cTnI, μg/l^#^	0 (0, 0)	0.69 (0, 0.03)	0.29 (0, 0.05)	
IL-18	742.92 (570.89, 868.93)	638.56 (247.47, 895.48)	514.77 (248.99, 637.92)	<0.05^c^
IL-1β	4.34 (2.82, 3.64)	3.84 (2.49, 3.81)	3.99 (2.59, 4.37)	
IL-6, pg/ml	9.08 (2.37, 4.51)	27.62 (2.77, 14.49)	25.63 (2.85, 9.49)	<0.05^a^
hs-CRP, mg/l^#^	2.08 (0.38, 1.21)	5.19 (0.69, 3.845)	3.79 (1.02, 3.93)	
TNF-α,pg/mL^#^	11.77 (2.32, 17,97)	36.72 (15.33, 51.54)	35.03 (15.65, 35.35)	<0.001^a^^c^

# *median (IQR)*,

* *mean ± SD*,

$ *n (%). Continuous, normally distributed variables among the four groups were analyzed by a one-way analysis of variance. The Kruskal-Wallis H- test was applied for data of this type that were not normally distributed. Continuous, normally distributed variables between two groups were analyzed by Student's t-test. The Mann Whitney U test was applied for data of this type that were not normally distributed. Categorical variables were compared by the χ2 test. NA, not available. Drinking history is defined as patients who consumed ≥ 50 g of alcohol per day*.

a *P. < 0.05 for equality between HC vs. CHD*.

b *P < 0.05 for equality between CHD vs. CHD-NAFLD*.

c *P < 0.05 for equality between HC vs. CHD-NAFLD*.

### CAD Patients Combined With NAFLD Had Worse Clinical Prognosis

Follow-up study was conducted for the 108 patients, and the median follow-up time was 2.16 (IQR: 2.04–2.24) years. A composite endpoint was observed in 10 out of 27 CAD-NAFLD patients (37.03%) and in 15 out of 81 CAD patients (18.52%). Binary logistic analyses demonstrated that complications of NAFLD were associated with an increased risk of composite endpoints [odds ratio (OR) = 2.059, 95% CI: 1.09–3.88, *P* = 0.033] after adjusting for confounding factors, including smoking history, male sex, age, drinking history, T2DM, HTN, abnormal thyroid function, OAD use and antihypertensive drug use (Supplementary Figure 1C). These results indicated that the CAD-NAFLD group presented a relatively worse prognosis than the CAD group. It is worth mentioning that the difference in baseline information of diseased groups was inconspicuous but the prognosis of CAD-NAFLD patients was significantly worse than CAD patients without NAFLD. We speculated that there were driving forces for cardiovascular events concealed under the complication of NAFLD.

FIGURE0

### Serum Metabolomic Features Are Changed in CAD Patients Combined With NAFLD

**Figure 1:**
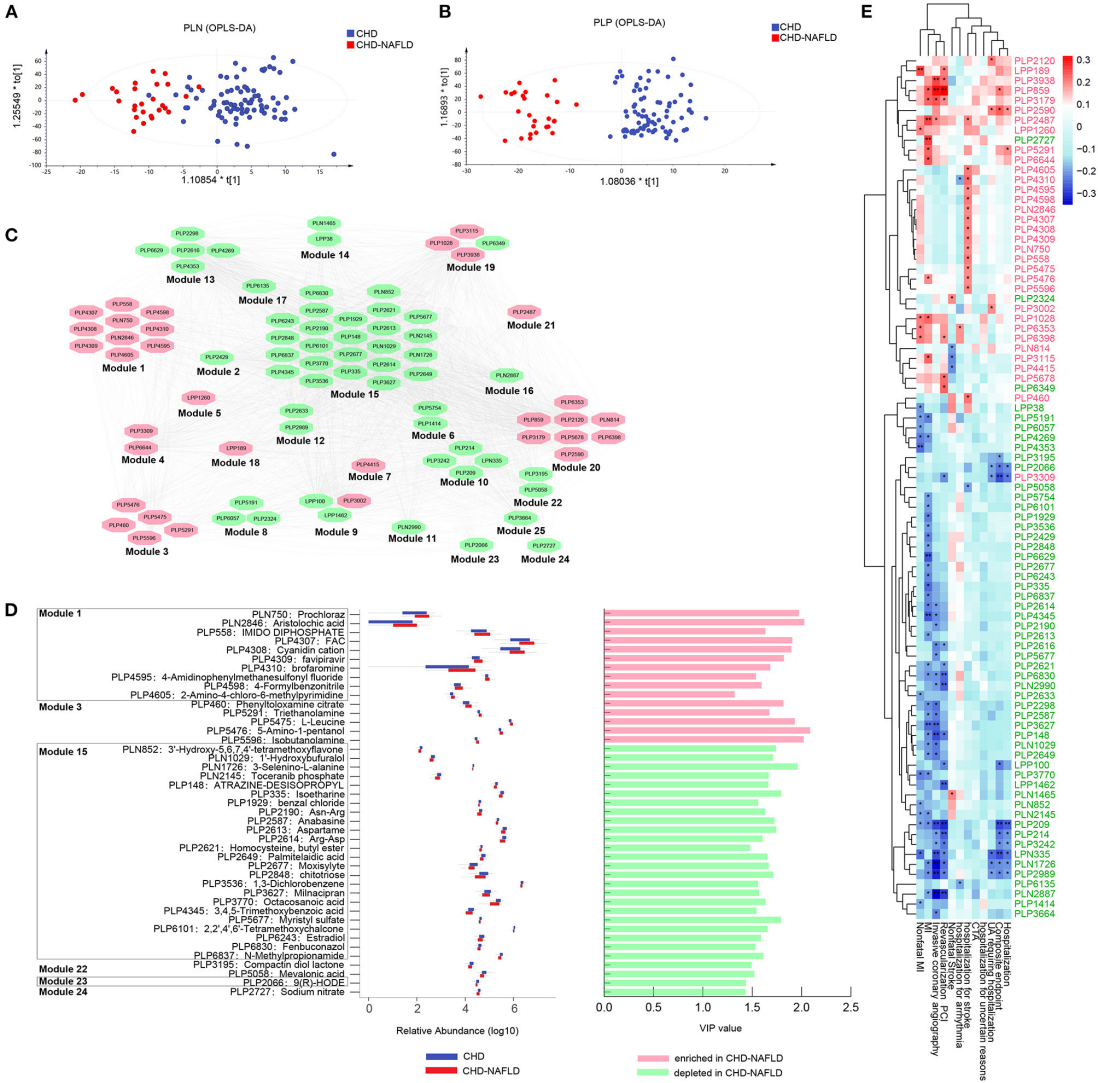
Serum metabolites were significantly changed and correlated with poor clinical outcomes in CHD-NAFLD patients. **(A,B)** Separation of serum metabolome between patients with CHD-NAFLD and patients with CHD under polar ionic negative mode and polar ionic positive mode, revealed by orthogonal partial least squares discrimination analysis (OPLS-DA). **(C)** Distinct serum metabolites cluster analysis by Pearson correlation. Red ovals represent metabolic modules that contains metabolites that are enriched in CHD-NAFLDs. Green ovals are metabolic modules that contains metabolites depleted in CHD-NAFLD. **(D)** Relative abundance and variable importance in projection (VIP) value of several distinct serum metabolites annotated by Human Metabolites Data Base (HMDB). Blue box: relative abundance in CHD group. Red box: relative abundance in CHD-NAFLD group. The bars on the right show enrichment (highlighted in pink) or depletion (highlighted in green) of specific serum metabolites in CHD-NAFLD group when compared with CHD group. The VIP values are also presented. **(E)** Spearman analysis of the covariant relationship between key metabolome and adverse cardiovascular events. Pink font: serum metabolites enriched in CHD-NAFLD. Green font: serum metabolites depleted in CHD-NAFLD. *P < 0.05, **P < 0.01.

Serum metabolites were analyzed by untargeted mass spectrometry (LC-MS). Four different types of metabolomic profiling modes—polar positive mode, polar negative mode, lipid positive mode, and lipid negative mode—yielded 14,585 (5,487 annotated), 7,394 (3,452 annotated), 5,193 (1,315 annotated), and 4,974 (701 annotated) metabolites, respectively, and the metabolites abundance profiles were obtained. We observed significantly different metabolomic profiles between CAD-NAFLD patients and CAD group under polar negative mode (Figure 1A) and polar positive mode (Figure 1B). Additional metabolite shifts were also discovered under lipid mode (Supplementary Figures 1A,B). The OR of all patient's smoking history, drug (OAD and HTN drug) history, sex, age, HTN, drinking history, DM, NAFLD, abnormal thyroid function was calculated, and all the features were not distinct among the groups except for NAFLD (*P* = 0.033) (Supplementary Figure 1C). After thresholding metabolites with a VIP value > 1 and a Wilcoxon rank-sum *P* value < 0.05, we filtered 87 annotated metabolites that significantly differed in abundance between CAD-NAFLD group and CAD group (Supplementary Table 1) and clustered them into 25 modules by Pearson correlation (Figure 1C). All serum metabolites contained in each module showed consistent alterations except for module 18 and module 19. Module 15 contained the largest number of metabolites and was a downregulated module. It is interesting to note that the metabolites contained in module 15, which was increased in CAD-NAFLD group, were mostly anti-inflammatory compounds (such as chitotriose, PLP2848, and estradiol, PLP6243). We also identified downregulated cardioprotective compounds in module 22 (e.g., mevalonic acid, PLP5058), module 23 [e.g., 9(R)-HODE, PLP2066], and module 24 (e.g., sodium nitrate, PLP2727) in CAD-NAFLD patients. Moreover, the enriched compounds in CAD-NAFLD group were mainly observed in three metabolite clusters including module 1, module 3, and module 20. To be noted that, these enriched compounds in module 1 and module 3 are known for their toxic effects (e.g., aristolochic acid, PLN2846, module 1; triethanolamine [TEA], PLP5291, module 3) or are considered allergic substances (isobutanolamine, PLP5596, module 3; phenyltoloxamine citrate, PLP460, module 3). The alteration of these crucial metabolites in different patient groups are presented in Figure 1D. In summary, CAD-NAFLD-associated serum metabolome alterations are characterized by the accumulation of toxic and proinflammatory compounds and scarcity of cardioprotective substances.

Furthermore, we analyzed the relationship between the alterations of serum metabolites and the clinical outcomes of patients. Spearman correlation showed that all the enriched serum metabolites were positively associated with non-fatal MI, MI events, invasive coronary angiography, revascularization percutaneous transluminal coronary intervention (PCI), and hospitalization for stroke (especially those in module 1), and the downregulated metabolites were negatively associated with hospitalization, composite endpoint, MI event, and non-fatal MI (Figure 1E). These results further confirmed that alterations in the serum metabolome are important driving factors in the prognosis of CAD-NAFLD patients. In addition, we further investigated the pathways these metabolites gathered on. Hypergeometric tests and topological data analysis (TDA) showed that terpenoid backbone biosynthesis was the most impacted pathway (Supplementary Figure 1D). Other impacted pathways included valine, leucine and isoleucine biosynthesis; valine, leucine and isoleucine degradation; aminoacyl-tRNA biosynthesis; and steroid hormone biosynthesis (Supplementary Figure 1D). In general, the accumulation of cardio-toxic and proinflammatory compounds and scarcity of cardioprotective substances in CAD-NAFLD patients are significantly associated with patient's prognosis and may affect clinical outcomes by regulating relevant pathways.

### Gut Microbiome Composition Is Changed in CAD Patients Combined With NAFLD and Associates With Serum Metabolome Alterations

Since some of the critical metabolites are associated with bacteria either by inhibiting bacterial enzymes (i.e., 4-Cyanobenzaldehyde, PLP4598, module1) or degraded by bacteria (i.e., sodium nitrate, PLP2727, module24), we speculate that the metabolome changes in patients with CAD-NAFLD may be mediated by the gut microbiota. We analyzed the gut microbiome by 16S rRNA sequencing. In the present microbiome investigation, a total of 2,830,519 high-quality 16S rRNA reads were obtained, with a median read count of 20,597 (range: 10,048 to 40,210) per 25 samples. A total of 626 OTUs were obtained after clustering. The rarefaction curves (Supplementary Figure 2A) of all samples supported the adequacy of the sequencing depth. In terms of alpha diversity, we observed no significant differences in the richness index (Supplementary Figure 2B) among the three groups. To assess the overall structures of the gut microbiota, a PCoA score plot based on the weighted UniFrac distances (Supplementary Figure 2C) was constructed. A Venn diagram was created to show the OTUs in each group (Supplementary Figure 2D).

**Figure 2:**
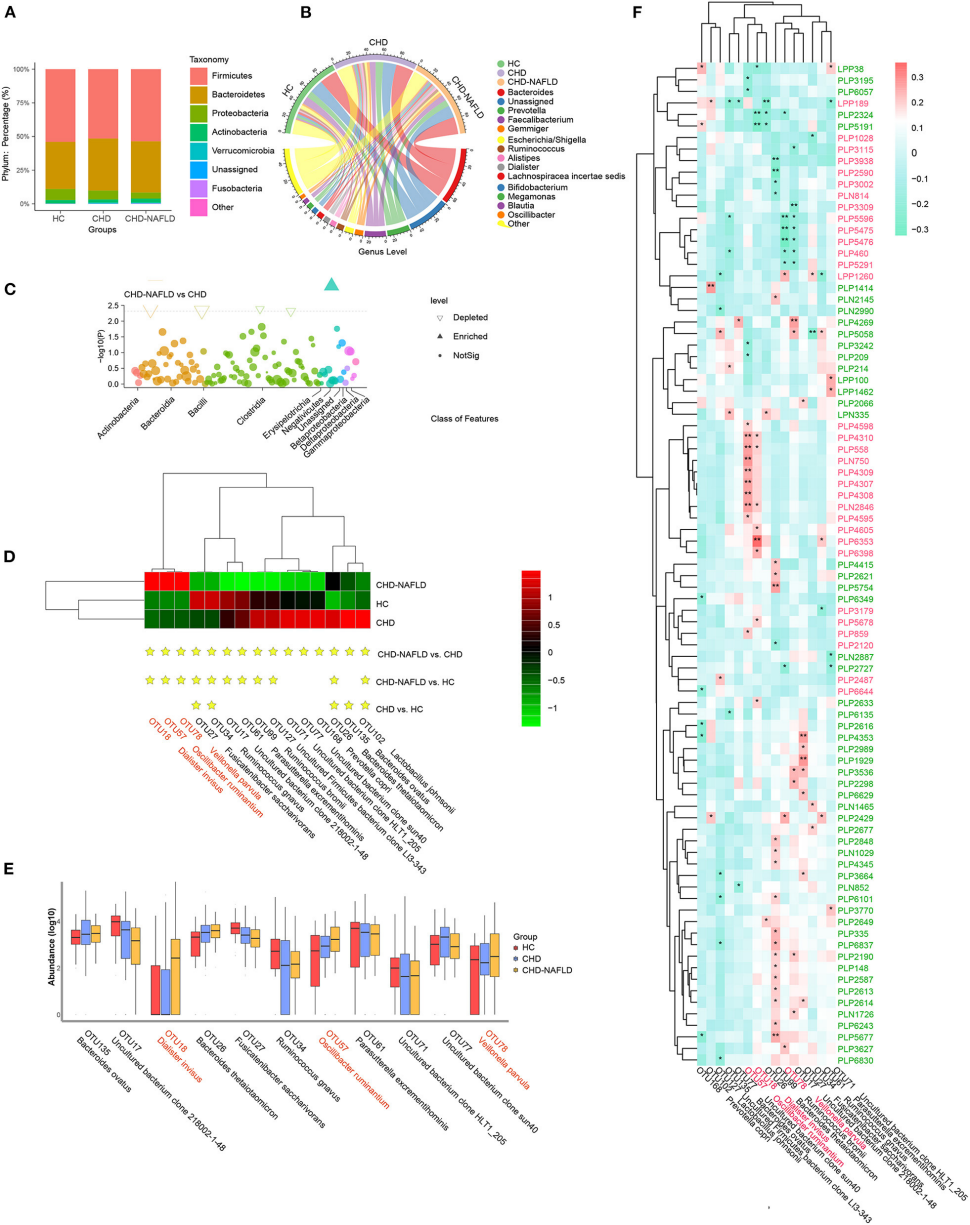
Distinct gut microbiome in different groups. **(A,B)** Phylum-level and genus-level composition of gut microbiome in patients with CHD-NAFLD, patients with CHD, and healthy controls (HC). **(C)** Differential bacteria at class-level between CHD-NAFLD group and CHD group. **(D)** Species-level alterations in gut microbiome in CHD-NAFLD, CHD, HC groups. Stars are marked for significant enrichment or depletion either in the CHD-NAFLD group, CHD group, or in the HC group. **(E)** The summed relative abundances of gut microbes were compared between the CHD-NAFLD and CHD groups based on species-level. **(F)** Spearman correlation between key gut microbiomes and altered serum metabolites. Red font: gut microbiomes enriched in CHD-NAFLD. Black font: gut microbiomes depleted in CHD-NAFLD. Pink font: serum metabolites enriched in CHD-NAFLD. Green font: serum metabolites depleted in CHD-NAFLD. *P < 0.05, **P < 0.01.

Gut microbial analysis revealed that although the change in alpha diversity was inconspicuous, the taxonomic composition of the microbiome of CAD-NAFLD patients differed from those of the microbiomes of CAD patients and HCs. Among all the identified OTUs, *Firmicutes* and *Bacteroidetes* were the two most abundant phyla (Figure 2A), and *Bacteroides, Prevotella*, and *Faecalibacterium* were the main genera in the gut microbiota (Figure 2B). At the class level, *Actinobacteria, Bacteroidia, Bacilli, and Clostridia* were decreased in CAD-NAFLD patients compared with CAD patients (Figure 2C). A total of 15 species were significantly different between CAD and CAD-NAFLD patients (Figure 2D), and their relative abundances are presented in a boxplot (Figure 2E). *V. parvula* (OTU78, *P* = 0.017), *D. invisus* (OTU18, *P* = 0.005), and *O. ruminantium* (OTU57, *P* = 0.045) were significantly increased in CAD-NAFLD patients compared with CAD patients, and *V. parvula* (OTU78) and *D. invisus* (OTU18) were identified as driver species in cardiovascular disease (20, 29–31). We also noticed that *B. ovatus* (OTU135, *P* = 0.004) and *Ruminococcus bromii* (OTU99, *P* = 0.005) were decreased in the CAD-NAFLD group compared with the CAD group. *R. bromii* (OTU99) was reported to have a protective effect on the heart and vessels by inhibiting DCA biotransformation and to have a hypoglycemic effect (32). *B. ovatus* (OTU135) reduces trinitrobenzene sulfonic acid-driven colonic inflammation (33). Our observation was consistent with these studies, suggesting that inflammation-promoting species were enriched in CAD-NAFLD, while anti-inflammatory species were decreased in CAD-NAFLD patients when compared to CAD patients without NAFLD.

We further correlated these microbiomes with key serum metabolites discovered previously, and the results were excellently consistent. The downregulated compounds were positively associated with the bacteria with decreased abundances, and the enriched compounds were positively associated with enriched taxa in the microbiomes (Figure 2F). In detail, enriched metabolites such as *prochloraz* (PLN750, module 1), *imido diphosphate* (PLP558, module 1), brofaromine (PLP4310, Module 1) were all significantly and positively correlated with *O. ruminantium* (OTU57, enriched in CAD-NAFLD). Other enriched metabolites, such as aristolochic acid, were significantly and positively correlated with *D. invisus* (OTU18, enriched in CAD-NAFLD). The remaining enriched metabolites, 5-amino-1-pentanol (PLP5476, module 3), TEA (PLP5291, module 3), isobutanolamine (PLP5596, module 3), and phenyltoloxamine citrate (PLP460, module 3), were all negatively correlated with uncultured bacterial clone 218002-1-48 (OTU17, decreased in CAD-NAFLD). For the downregulated metabolites, *estradiol* (PLP6243, module 15) and 2,2',4',6'-tetramethoxychalcone (PLP6101, module 15) were significantly and positively correlated with *R. bromii* (OTU99, decreased in CAD-NAFLD), and moxisylyte (PLP2677) was positively and significantly correlated with *R. gnavus* (OTU34, decreased in CAD-NAFLD). Additional details are shown in Figure 2F. It is not difficult to conclude that the accumulation of toxins was associated with increases in potentially pathogenic microbiomes and with decreases in potentially beneficial microbiomes, while reduction in cardioprotective compounds were associated with depletion of potentially beneficial microbiomes.

### Variations in Gut Microbiome Composition Lead to Altered Metabolic Pathway in CAD-NAFLD Patients

**Figure 3:**
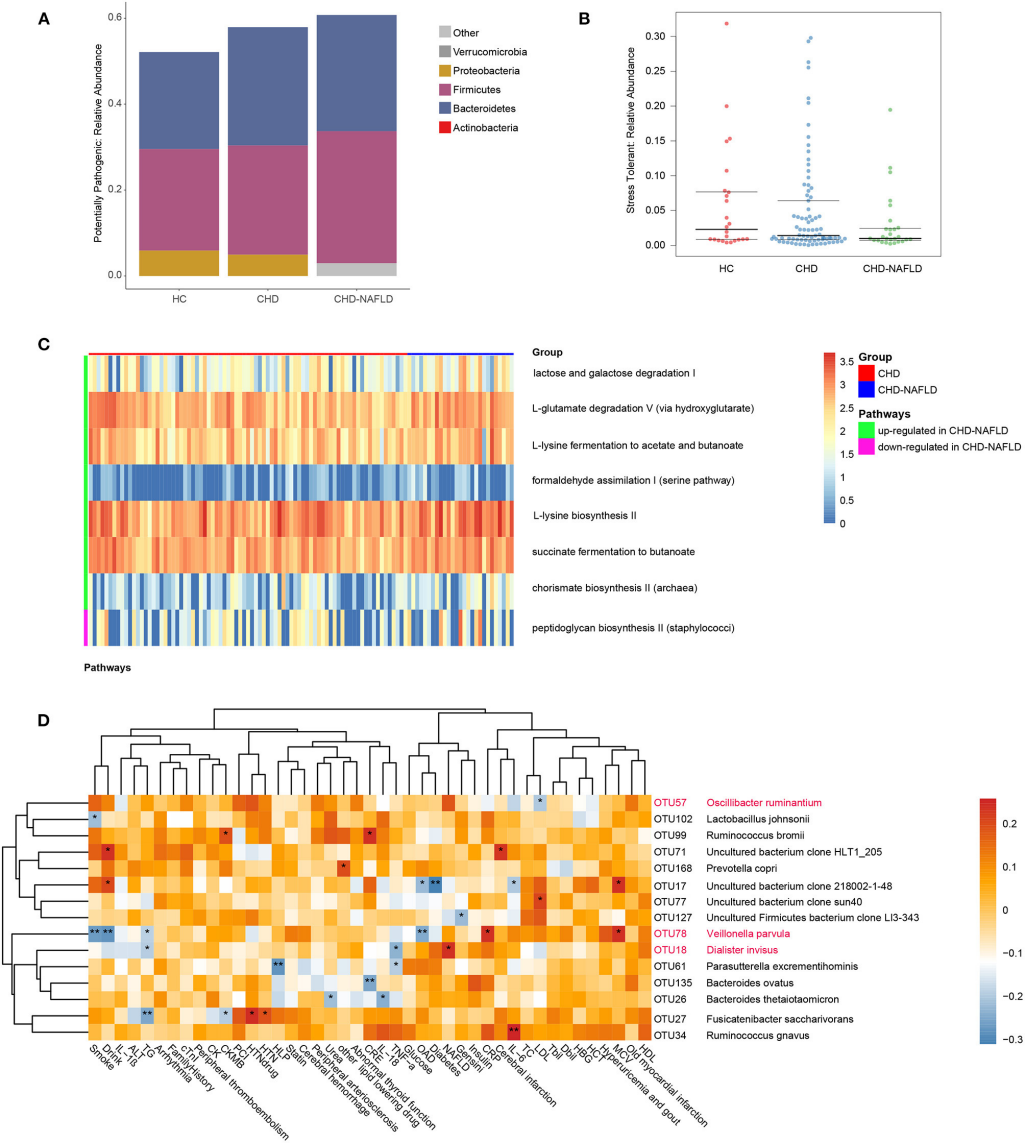
Function prediction of gut microbiome among three groups at OTU level. **(A,B)** Predicted high-level phenotypes (potential pathogens and stress tolerant), calculated by BugBase. **(C)** Relative activity of pathways in CHD and CHD-NAFLD. **(D)** Correlation of the CHD-NAFLD featured gut microbes with clinical indexes. Rows: CHD-NAFLD-positive OTUs are highlighted in red, and CHD-NAFLD-negative OTUs are in black. Columns: clinical indexes. *P < 0.05, **P < 0.01.

To further substantiate the potential role of gut microbes in the alteration of the serum metabolome, we focused on the microbial rRNAs predicted to correspond to microbes with known metabolic functions. A total of 626 OTU data points were functionally annotated with PICRUSt2 software. The proportion of potential pathogens showed a gradual increasing trend HCs, CAD patients, and CAD-NAFLD patients (Figure 3A). Although there isn't significant difference in HCs and CAD patients, the proportion of potential pathogens is significantly higher in CAD-NAFLD patients when compared with HCs (*P* = 0.039), representing a characteristic disorder of the gut microbiota. Furthermore, the abundances of stress-tolerant bacteria showed a decreasing trend in HCs, CAD patients, and CAD-NAFLD patients (Figure 3B). The enrichment of potential pathogens and decreases in stress-tolerant bacteria may be one of the reasons for the worse prognosis of CAD-NAFLD.

The impacted metagenomic pathways were predicted using the PICRUSt2 tool based on the MetaCyc database. A total of 8 pathways were found to differ in activity level between the CAD group and the CAD-NAFLD group (Figure 3C). We identified 7 upregulated pathways (lactose and galactose degradation I, formaldehyde assimilation I (serine pathway), L-lysine biosynthesis II, chorismate biosynthesis II from Archaea, L-glutamate degradation V via hydroxyglutarate, L-lysine fermentation to acetate and butanoate, succinate fermentation to butanoate). Only one pathway was downregulated. We speculate that the changes in these pathways may be the cause of the alterations in the metabolome. The gut microbiome may affect the metabolome by up- or downregulating these pathways. Moreover, these pathways are mainly amino acid-associated pathways and glucose-associated pathways, which is consistent with the pathways associated with the identified crucial metabolites (Supplementary Figure 1D). These differences in pathways active level further support the strong association between the gut microbiome and serum metabolome alterations in CAD-NAFLD patients.

Given that the gut microbiome is associated with prognosis-related serum metabolites, it is not hard to presume that these gut microbiomes also affect the prognosis of CAD-NAFLD patients. Correlations between CAD-NAFLD-associated OTUs and clinical indexes were calculated by Spearman correlation (Figure 3D). We noted that *V. parvula (OTU78)*, a species enriched in CAD-NAFLD, was positively correlated with C-reactive protein (CRP), an inflammation factor. We also observed that species that were decreased in CAD-NAFLD, such as *uncultured bacterium clone 218002-1-48* (OTU17), *Parasutterella excrementihominis* (OTU61), and *Bacteroides thetaiotaomicron* (OTU26), were negatively correlated with inflammatory factors (e.g., IL-6, TNF-α, and IL-26). Moreover, the gut bacteria with decreased abundances (*B. ovatus*, OUT135; *F. saccharivorans*, OTU27; *uncultured Firmicutes bacterium clone LI3-343*, OTU127) were also negatively correlated with markers that represent the severity of the disease, such as Gesini score, CK-MB (creatine kinase MB), and creatine. These correlations further showed the association between the decreased beneficial species and higher risk of cardiovascular disease.

### Alterations of Bacterial Species and Associated Metabolome Changes Are Related to Adverse Clinical Outcomes in CAD Patients Combined With NAFLD

**Figure 4:**
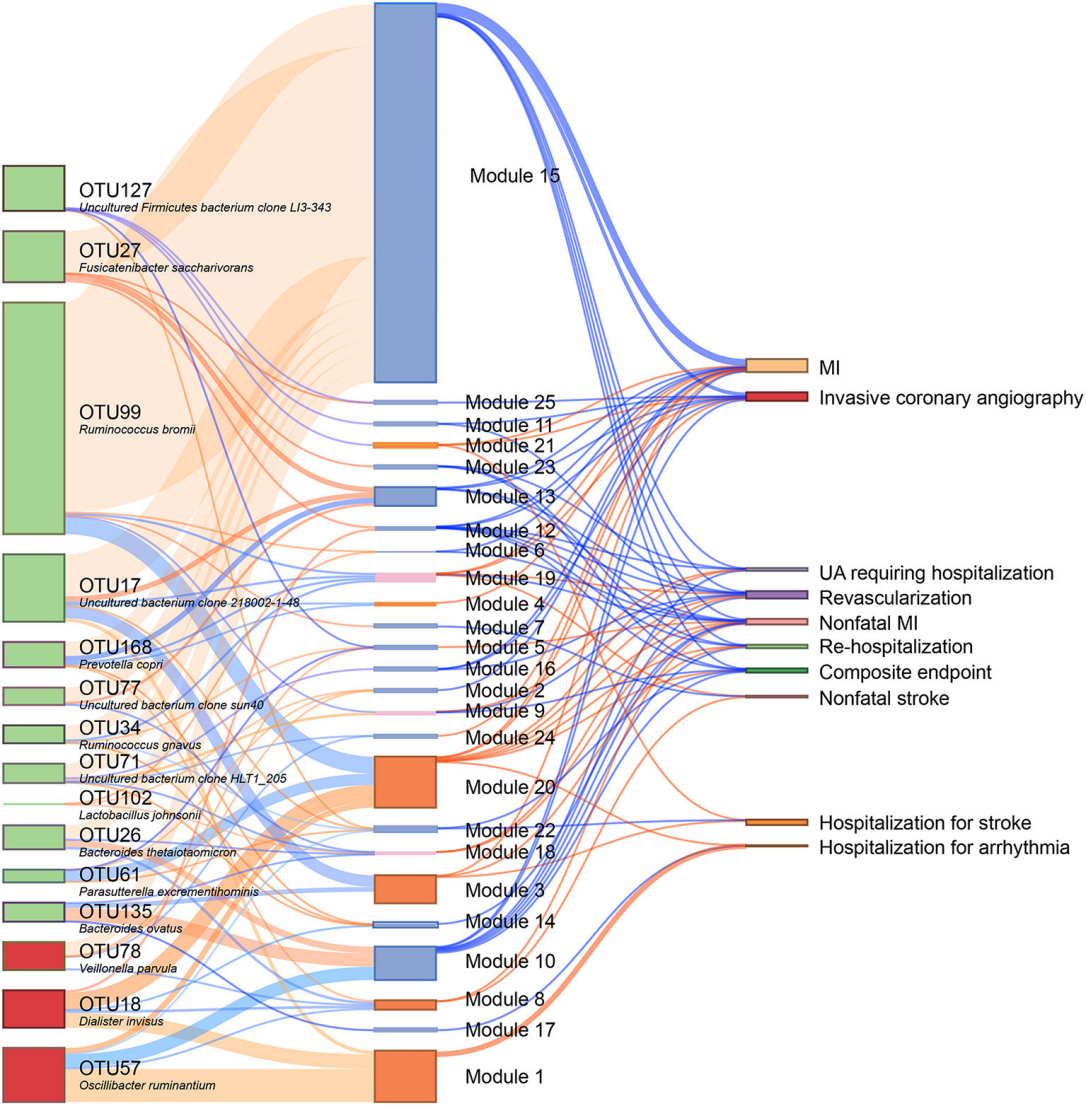
Interrelationship between CHD-NAFLD-associated flora, serum metabolic features and clinical outcomes. Red connections indicate positive correlations, and blue connections indicate negative correlations (Spearman correlation analysis, *P* < 0.05). In the left first column, green boxes indicate CHD-NAFLD-negative OTUs, and red boxes indicate CHD-NAFLD-positive OTUs. In the middle column, light blue boxes indicate CHD-NAFLD-negative metabolic features, and orange boxes indicate CHD-NAFLD-positive metabolic features. A composite endpoint is defined as all-cause mortality and/or reoccurrence of ACS and/or readmission for cardiac causes.

**Figure 5:**
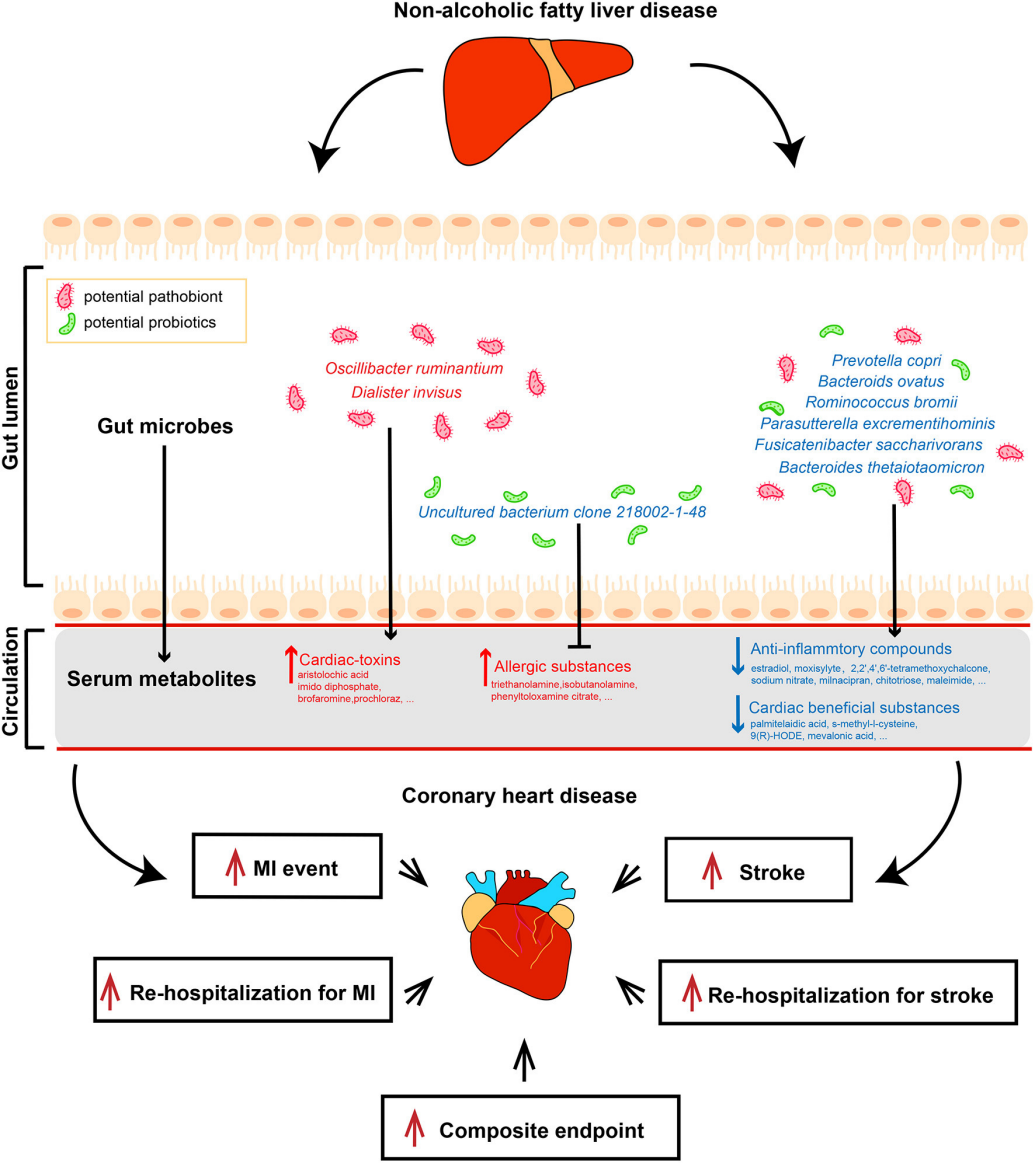
Multi-omics analysis illustrates the interrelationship between non-alcoholic fatty liver disease and coronary heart disease. Features colored in red represent increase in corresponding gut microbiome or serum metabolites. Features colored in blue represent depletion in corresponding gut microbiome or serum metabolites. Arrows represent significant correlations between features.

We subsequently analyzed the correlations of serum metabolites with the gut microbiome and with CAD-NAFLD patient prognosis, which were visualized in Sankey plots (Figure 4). In general, at the species level, *V. parvula* (OTU78), *D. invisus* (OTU18), and *O. ruminantium* (OTU57) were significantly enriched, while *P. excrementihominis* (OTU61), *B. ovatus* (OTU135)*, F. saccharivorans* (OTU27), *R. gnavus* (OTU34), *uncultured bacterium clone 218002-1-48* (OTU17)*, P. copri* (OTU168) and *R. bromii* (OTU99) were decreased. The serum metabolites shown in Figure 4 were mostly decreased in CAD-NAFLD patients compared with CAD patients, while metabolites in module 1, 3, 8, 20, and 21 were enriched. Two metabolite clusters (module 10 and 15) were both composed of decreased metabolites and positively correlated with decreased bacteria (OTU17, OTU26, OTU77, OTU71, OTU127, OTU135, OTU61, OTU27, OTU34, OTU99, OTU102, and OTU168) or negatively correlated with enriched bacteria (*O. ruminantium*, OTU57). Additionally, another 2 metabolite clusters (module 3 and 20) were composed of enriched metabolites, which were either positively correlated with enriched bacteria (OTU18 and OTU57) or negatively associated with decreased bacteria (OTU135, OTU17, and OTU99). Moreover, the enriched metabolites were highly associated with a higher risk of MACEs, while the decreased metabolites were negatively associated with poor clinical outcome. This result systematically and clearly illustrated that the alteration of the serum metabolome in CAD-NAFLD is highly associated with poor clinical outcome through toxin accumulation and decreased protection. The relationship between NAFLD and CAD is illustrated in Figure 5 in a more intuitive manner. This multiomics study highly suggests that complications of NAFLD are associated with gut microbiome alterations, such as the enrichment of *D. invisus* (OTU18) and *O. ruminantium* (OTU57) and the decrease in *P. copri* (OTU168). The change in gut microbes was further associated with serum metabolome alterations, and cardiac toxins (e.g., *aristolochic acid, prochloraz*, and *imido diphosphate*) and allergic compounds (e.g., *isobutanolamine* and *phenyltoloxamine citrate*) accumulated in the circulation, while anti-inflammatory compounds (e.g., *estradiol, 2,2',4',6'-tetramethoxychalcone, moxisylyte, sodium nitrate, milnacipran*, and *chitotriose*) and cardioprotective substances (e.g., *mevalonic acid, 9(R)-HODE, S-methyl-L-cysteine*, and *palmitelaidic acid*) were decreased. These metabolome changes were highly associated with composite endpoints, MI events, and rehospitalization in CAD-NAFLD patients. In conclusion, our multiomics study presents associations among NAFLD, the gut microbiome, the serum metabolome, and CAD patient prognosis.

## Discussion

In recent years, accumulating evidence has shown that changes in the microbiota are associated with CAD progression (34–36). NAFLD is considered a powerful driving force in CAD since fatty liver is strongly associated with systemic inflammation (37, 38). The regulation of the gut microbiota and its bacterial products plays an important role in the development of NAFLD (39–41). Intestinal bacterial species and their metabolites regulate adipose tissue and intestinal homeostasis and then contribute to the pathogenesis of NAFLD. Given the notably strong association between NAFLD and CAD, we demonstrate in this work that the gut microbiome in CAD individuals complicated with NAFLD has functional potential for accelerated biosynthesis of a number of inflammatory compounds, leading to elevated serum concentrations of proinflammatory metabolites and depleted potential cardiac-beneficial compounds, and finally resulting in aggravated adverse cardiovascular events.

We paid special attention to several microbiome associated-metabolites and modules that were significantly associated with poor clinical outcome. Prochloraz (PLN750, module 1) was significantly increased in CAD-NAFLD patients and was positively correlated (*P* = 0.003, Cor = 0.284) with *O. ruminantium* (OTU57). Prochloraz was documented as a gut microbiome-associated hepatotoxic metabolite and affects glucolipid metabolism (42, 43). Moreover, brofaromine (PLP4310, module 1), a MAO inhibitor, showed a positive correlation (*P* = 0.008, Cor = 0.253) with *O. ruminantium* (OTU57). Aristolochic acid (PLN2846, module 1), a well-known nephrotoxic phytochemical that was reported to induce heart failure in zebrafish embryos (44), was also significantly increased in NAFLD patients and was positively correlated (*P* = 0.036, Cor = 0.202) with *D. invisus* (OTU18). Additionally, *O. ruminantium* (OTU57) and *D. invisus* (OTU18) were shown to be greatly enriched in patients with atherosclerotic cardiovascular disease compared with healthy people (45, 46). Additionally, imido diphosphate (PLP558, module 1) was positively correlated (*P* = 0.042, Cor = 0.196) with *D. invisus* (OTU18). Our results suggest that potential pathogens such as *O. ruminantium* (OTU57) and *D. invisus* (OTU18) are significantly enriched in CAD-NAFLD and associated with the elevation in toxic serum metabolites (e.g., prochloraz and aristolochic acid), and the above-mentioned metabolites (prochloraz, brofaromine, aristolochic acid, imido diphosphate) are all positively associated (*P* < 0.05) with rehospitalization for stroke in CAD-NAFLD patients. Besides *O. ruminantium* (OTU57) and *D. invisus* (OTU18), *V. parvula* (OTU78) was also elevated in CAD-NAFLD, and it was documented to be enriched in rheumatoid arthritis (47) and Crohn's disease (48). *V. parvula* (OTU78), as a nitrate-reducing bacteria (49), was observed to be negatively associated (*P* = 0.025, Cor = −0.215) with sodium nitrate (PLP2727, module 24), which attenuates inflammation in metabolic syndrome patients (50) and is depleted in CAD-NAFLD patients. To sum up, our results suggested that the enrichment of potential pathogens (*O. ruminantium, D. invisus, V. parvula*) in the gut microbiome associates with increases in toxic metabolites (e.g., prochloraz and aristolochic acid) and decreases in anti-inflammatory metabolites (e.g., sodium nitrate), and this alteration in serum metabolome strongly associates with worse clinical outcomes in CAD-NAFLD patients. This association is suggestive to the mechanism of the progression of CAD-NAFLD. We speculate that accumulation of potential pathogenic gut microbiome in CAD-NAFLD is at least one of the important driving forces in disease progression.

TEA (PLP5291, module 3) caught our special attention, as it was enriched in CAD-NAFLD patients and significantly correlated with bacterial clone 218002-1-48 (OTU17, *P* = 0.024, Cor = −0.217) and shows negatively correlation trend with *P. excrementihominis* (OTU61, Cor = −0.028). TEA was documented to cause susceptibility to pathogenic infection by inducing apoptosis (51) and can be degraded by *Alcaligenes faecalis* (52). *Alcaligenes faecalis* belongs to the *Burkholderiales* order and is associated with atherosclerotic cardiovascular disease (45). Consistently, we observed that *P. excrementihominis* (OTU61), which also belongs to the *Burkholderiales* order, was depleted in CAD-NAFLD. Thus, the less harmful TEA underwent biodegradation and more accumulated in serum. Moreover, isobutanolamine (PLP5596, module 3) and phenyltoloxamine citrate (PLP460, module 3), both of which cause contact allergies (53), were also negatively correlated with uncultured bacterium clone 218002-1-48 (OTU17, *P* < 0.05). Consistently, these compounds were all positively associated (*P* < 0.05) with rehospitalization for stroke. In addition to these three compounds, the remaining two compounds in module 3 were also negatively associated with uncultured bacterium clone 218002-1-48 (OTU17, *P* < 0.05). These results suggested that the depletion of uncultured bacterium clone 218002-1-48 (OTU17) is associated with the accumulation of toxic metabolites (e.g., isobutanolamine and phenyltoloxamine citrate) in CAD-NAFLD patients, and we speculate that uncultured bacterium clone 218002-1-48 (OTU17) may play an inhibitory role in the biosynthesis of these toxic metabolites, just as *Alcaligenes faecalis does to TEA*. Combined with the results mentioned in the previous paragraph, it is worth mentioning that the increase of potential gut microbiome and decrease of potential beneficial gut microbiome modulate the serum metabolome to a more toxic status.

Not only did we identify the accumulation of toxic metabolites, we also found that a large number of protective metabolites were depleted in CAD-NAFLD patients, and these beneficial metabolites were mainly clustered in module 15. Estradiol (PLP6243, module 15) is one of the potentially beneficial substances that was decreased in CAD-NAFLD patients and was observed to be positively correlated (*P* = 0.027, Cor = 0.213) with *R. bromii* (OTU99, decreased in CAD-NAFLD). Estradiol (PLP6243, module 15) was shown to have protective effects against reperfusion injury (54) and to alleviate of cardiac aging caused by galactose (55). Additionally, 2,2',4',6'-tetramethoxychalcone (PLP6101, module 15) and chitotriose (PLP2848, module 15) are both anti-inflammatory metabolites (56, 57) that were decreased in CAD-NAFLD and associated with the decrease in *R. bromii* (OTU99). Moreover, other metabolites in module 15 also have anti-inflammatory effects, such as milnacipran (58) (PLP3627, module 15), palmitelaidic acid (PLP 2649, module 15), and moxisylyte (PLP2677, module 15). Palmitelaidic acid prevents cardiac fibrosis (59) and was documented to be decreased in CAD-NAFLD patients (60), which is consistent with our observation. It was associated (*P* = 0.038, Cor = 0.200) with the depletion of *B. thetaiotaomicron* (OTU26). Moxisylyte (PLP2677, module 15) was associated (*P* = 0.043, Cor = 0.195) with *R. gnavus* (OTU34) and were reported to protect against inflammatory attacks (61). It is worth mentioning that these decreased serum metabolites all negatively correlated (*P* < 0.05) with MI events in CAD-NAFLD patients. The remaining metabolites in module 15 were highly suggested to be protective against cardiac events since they presented consistent alteration and microbiome association Combined with the accumulation of toxic compounds mentioned previously, the decreases in these potentially beneficial metabolites further strengthened the toxic serum status in CAD-NAFLD patients.

Notable changes in the gut microbiota in individuals with NAFLD had a functional potential to accelerate the biosynthesis of the above toxic compounds and the depletion of cardiac-protective compounds, leading to a higher risk of inflammatory exposure and aggravated CAD clinical outcome (Figure 4). *D. invisus* (OTU18), one of the species enriched in NAFLD, is predicted to be associated with the production of toxins such as imido diphosphate (PLP558, module 1) and aristolochic acid (PLN2846, module 1). Consistently, it has been reported that *D. invisus* (OTU18) s associated with intestinal inflammation, such as chronic apical abscess (62), is increased in human milk-fed piglets (63) and is positively associated with osteoarthritis (64). Another important potential pathogenic species, *V. parvula* (OTU78), was predicted to be associated with severe hepatitis (65). Consistently, *V. parvula* (OTU78) was significantly increased in CAD-NAFLD and negatively associated with an anti-inflammatory compound (sodium nitrate, PLP2727) due to its degradation (49). In general, *D. invisus* (OTU18) and *V. parvula* (OTU78) are two well-known pathogens and is also considered as potential pathogens in our study since they are associated with accumulation of serum toxins. This further suggested that *D. invisus* (OTU18) and *V. parvula* (OTU78) are important driving forces in the CAD-NAFLD pathogenesis.

We paid special attention to *R. gnavus* (OTU34), a controversial species (66), which showed a gradual decrease in HCs, CAD patients, and CAD-NAFLD patients. *R. gnavus* (OTU34) was negatively associated with moxisylyte, an anti-inflammatory compound. Consistently, *R. gnavus* was found to be decreased in CAD-NAFLD (20, 67), and our observation further confirmed that *R. gnavus* (OTU34) may be protective in CAD. Additionally, *F. saccharivorans* gradually decrease in HCs, CAD patients, and CAD-NAFLD patients. Previous studies indicated that *F. saccharivorans* (OTU27) was significantly decreased in inflammatory disease and that *F. saccharivorans* (OTU27) was involved in short-chain fatty acid- (SCFA) production (64, 68) and produced lactic acid, formic acid, acetic acid and succinic acid as fermentation end products from glucose (69). Therefore, it was speculated that *F. saccharivorans* (OTU27) might be related to lipid metabolism alterations in CAD patients and to the even worse lipid metabolism alterations in CAD-NAFLD patients. A newly found species, uncultured bacterium clone 218002-1-48 (OTU17), was shown to have an irreplaceable role in the downregulation of protective serum metabolites. The compounds in module 3 (PLP5475, PLP5476, PLP5291, PLP5596, and PLP460) were all decreased in CAD-NAFLD and have been shown to have cardiotoxic effects. Moreover, all metabolites in module 3 were significantly associated with uncultured bacterium clone 218002-1-48 (OTU17). We speculated that the decrease in OTU17 reduced the inhibition of these toxins. Although OTU17 may be a newly discovered species, it is worthy of further study and may be a potential treatment for CAD-NAFLD patients. To sum up, the gradual decrease of *R. gnavus* (OTU34) and *F. saccharivorans* (OTU27) highly suggest they are potential probiotics. Additionally, the down-regulation of these potential probiotics played supporting role in CAD-NAFLD disease progression by down-regulating cardiac-protective compounds.

To explain the above interrelationships, we introduce a new concept: liver-gut microbiota-heart axis, as a possible mechanism under these interactions. On the one hand, the concept of liver-gut microbiota axis has already been proposed and confirmed by many researches (70, 71). Because of the direct connection via portal vein between the intestines and the liver, gut microbiota and associated dysbiosis have been known as regulators in the pathophysiology of NAFLD (71). Thus, the alterations in gut microbiota in CAD-NAFLD patients can be explained by liver-gut microbiota axis. On the other hand, gut-heart axis has emerged as a novel concept to provide new insights into the complex mechanisms of cardiovascular disease and offer new therapeutic targets (72, 73). Moreover, multi-point axis is not unusual in disease development. For instance, recent research work has presented a liver-brain-gut axis in inflammatory bowel disease (74). Thus, we come up with a new concept of liver-gut microbiota-heart axis and we believe this axis may be the possible underlying mechanism in the prognosis of CAD-NAFLD. However, more experiments are needed to prove the existence of this novel axis, as well as its possible regulation in disease.

This work illustrated that CAD-NAFLD patients has worse clinical outcome than patients with CAD alone. We discovered that protective metabolites are decreased and proinflammatory toxins are increased in the serum of CAD-NAFLD patients compared with CAD patients, and the changes in metabolites were closely associated with changes in the gut microbiome. Therefore, we speculate that the increase in pathogenic gut bacteria (e.g., *V. parvula, O. ruminantium*, and *D. invisus*) and the decrease in beneficial gut bacteria (e.g., *R. gnavus* and *F. saccharivorans*) lead to the depletion or accumulation of different serum metabolites and finally lead to a higher risk of cardiovascular events. We speculate that it is the complication of NAFLD that impacted the serum metabolome and further accelerated the pathogenic process of CAD. Since liver is an organ known for breaking down harmful substances and regulating most chemical levels in the blood (75, 76), the healthiness of liver strongly affects serum metabolome. NAFLD is the condition when too much fat is stored in liver cells, in other word, the liver is work-overload. In this case, the blood-regulating function of liver is significantly reduced and more harmful substances are accumulated in the blood (77). Moreover, research work has shown that hepatocyte affects bile acids, gut microbiota and metabolome contributing to regulate glucose and lipid metabolism (78). Experiments done in mice have found that the hepatocyte deletion of MyD88, a crucial gene in obesity and diabetes, induces changes of specific gut microbes (78). All these evidences supported that NAFLD contributes to the alteration in serum metabolome, and gut microbiota may accelerate this process. Further, we found that the NAFLD-induced metabolic change could be summarized into the accumulation of cardio-toxic and proinflammatory compounds and scarcity of cardioprotective substances. This alteration definitely affects CAD in many ways, such as the accumulation of lipids (21), sclerosis of arteries (79), and exacerbates the inflammatory response (80). Our results provide a new insight to the understanding of disease mechanism and we highly speculate that gut microbiome plays at least one important role in the progression of CAD-NAFLD. To our knowledge, this is the first multiomics analysis on the serum metabolome, gut microbiome, clinical indexes and outcomes in CAD-NAFLD patients and CAD patients. Critical metabolome and microbial changes associated with adverse cardiovascular events in CAD-NAFLD were explored through a multiomics study. Our work is also the first to provide a schematic hypothesis that could be used as a framework for proof-of-concept studies that link the impact of serum metabolome and gut microbiome alterations with CAD-NAFLD patient outcomes. These findings are in line with the results from other NAFLD and CAD microbiome studies. Moreover, besides the several metabolites we discussed, the rest metabolites clustered into the same modules as the discussed metabolites are highly speculated to play the same role, since all the metabolites in one module are consistent in their spearman correlation with gut microbiome as well as their alteration in serum concentration. Not only did we identified currently known serum metabolites that are highly likely to participate in disease progression, but also we sorted out a list of potentially disease-related metabolites that we currently know little about. Our study provided potential targets for CAD-NAFLD treatment.

Our study is meaningful to current medication and treatment of CAD-NAFLD patients. Current treatment of CAD-NAFLD patients mainly focused on the treatment of CAD and blood lipid level and lipid-lowering drugs are the current mainstream treatment for NAFLD. But if the treatment of gut microbiomes is taken into consideration, the prognosis of CAD-NAFLD patients may be much better since our study showed that gut microbiome plays a driving force in disease progression. In addition, we provided detailed list of CAD-NAFLD related gut microbiomes and serum metabolites, which could all become potential targets for treatment. Last but not least, our newly proposed “liver-gut microbiota-heart axis” also indicates the importance of liver's health in CAD patients. The health status of liver may affect the clinical outcomes of CAD patients by the interrelationship with gut microbiome.

Notably, this study has some limitations. First, it is a correlation study and did not include animal experiments on the function of the key differential bacteria. As few reports have been made about the metabolic function of the differential bacteria, we need to undertake additional studies to confirm the function of these bacteria. Second, considering that our study was a single-center study, additional multicenter studies are needed to confirm the results. Third, the study had a small sample size. An enlarged study population would make the results more convincing.

## Conclusions

Taken together, our results indicate that NAFLD may drive the progression of CAD by altering the gut microbiome. The changes in the intestinal gut microbiota further modulate the serum metabolome of CAD-NAFLD patients toward a worse status, i.e., reducing cardioprotective compounds such as estradiol, sodium nitrate, chitotriose, and maleimide and increasing potentially toxic and proinflammatory compounds such as triethanolamine, isobutanolamine, aristolochic acid, prochloraz, and imido diphosphate, thus leading to a higher risk of MACEs. We came up with a novel concept of “liver-gut microbiota-heart axis” which could possible explain this interrelationship. Our study presents a wide perspective to develop new diagnostic and therapeutic approaches, and additional comprehensive studies are desired. We also suggested the importance of liver's health in CAD patients.

## Data Availability Statement

The datasets presented in this study can be found in online repositories. The names of the repository/repositories and accession number(s) can be found in the article/Supplementary Material.

## Ethics Statement

The studies involving human participants were reviewed and approved by the Ethics Review Board at the Institute of Basic Medical Sciences, Chinese Academy of Medical Sciences. The patients/participants provided their written informed consent to participate in this study.

## Author Contributions

SZ and XH: concept, design, and study supervision. XH and RZ: clinical research, analysis, and interpretation of data. RZ, XH, HL, YS, XZ, and YF: sample and data acquisition. RZ, XH, and SZ: writing, review, and/or revised the manuscript. All authors read and approved the final manuscript.

## Funding

This work was supported by the Beijing Natural Science Foundation No. 7202152, the CAMS Innovation Fund for Medical Sciences (CIFMS) (Nos. 2017-I2M-2-002, 2016-I2M-1-002, 2017-I2M-2-001, and 2019-I2M-1-001), the National Key Research and Development Program of China (Grant No. 2016YFC0901502), and Center for Rare Diseases Research, Chinese Academy of Medical Sciences, Beijing, China (Grant No. 2016ZX310174-4).

## Conflict of Interest

The authors declare that the research was conducted in the absence of any commercial or financial relationships that could be construed as a potential conflict of interest.

## Publisher's Note

All claims expressed in this article are solely those of the authors and do not necessarily represent those of their affiliated organizations, or those of the publisher, the editors and the reviewers. Any product that may be evaluated in this article, or claim that may be made by its manufacturer, is not guaranteed or endorsed by the publisher.

## Glosarry

HCs, Healthy controls; CAD, Coronary artery disease; CAD-NAFLD, Coronary artery disease complicated with fatty liver disease; BMI, Body mass index; BUN, Blood urea nitrogen; CK, Creatine kinase; CR, Creatinine; cTnI, Cardiac troponin I; HDL-C, High-density lipoprotein cholesterol; hsCRP, High-sensitivity C-reactive protein; LC-MS, Liquid chromatography-mass spectrometry; LDL-C, Low-density lipoprotein cholesterol; MI, Myocardial infarction; No. of SV, Number of stenosed vessels; OAD, Oral antidiabetic drugs; OPLS-DA, Orthogonal projection to latent structure-discriminant analysis; OUT, Operational taxonomic unit; PCI, Percutaneous transluminal coronary intervention; SBP, Systolic blood pressure; TG, Triglyceride; TNF-α, Tumor necrosis factor alpha; VIP, Variable importance in projection; T2DM, type 2 diabetes mellitus; HTN, hypertension; γ-GGT, γ-glutamyl transferase; MACEs, major adverse cardiac events; CV, coefficient of variation; QC, quality control; PICRUSt2, Phylogenetic Investigation of Communities by Reconstruction of Unobserved States; TEA, triethanolamine; TDA, topological data analysis; SCFA, short-chain fatty acid.
