# Supplementary material for: Alterations of Gut Microbiome and Serum Metabolome in Coronary Artery Disease Patients Complicated With Non-alcoholic Fatty Liver Disease Are Associated With Adverse Cardiovascular Outcomes
Source: Front Cardiovasc Med. 2022 Jan 3;8:805812. doi: 10.3389/fcvm.2021.805812 (PMC8761954; doi:10.3389/fcvm.2021.805812)
Supplement: Supplementary file 1 [file Data_Sheet_1.PDF]

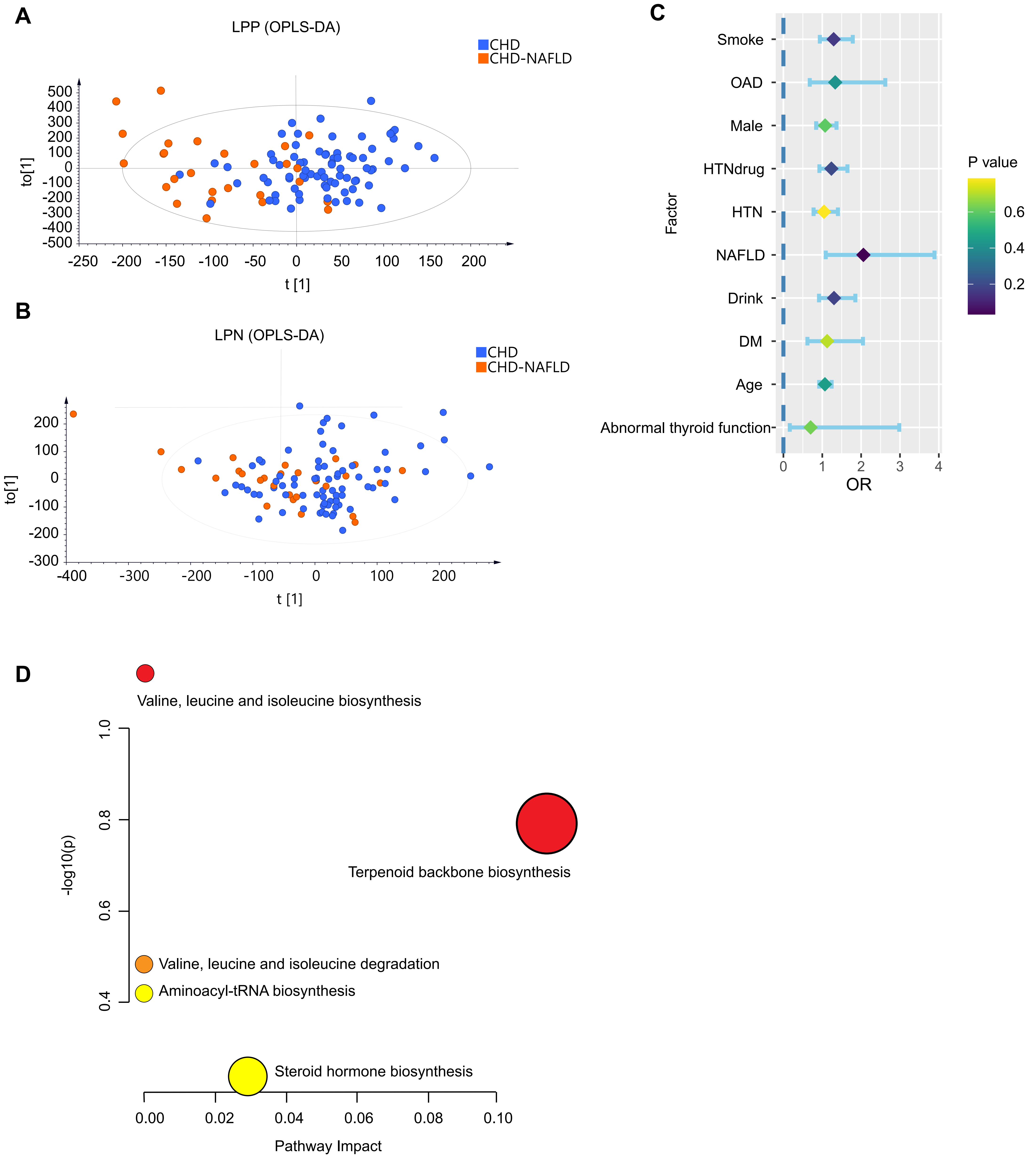

Figure S1. Other key metabolites discovered in patients with CHD-NAFLD. (A-B) Separation of serum metabolome between patients with CHD-NAFLD and patients with CHD under lipid positive mode and lipid negative mode, revealed by orthogonal partial least squares discrimination analysis (OPLS-DA). **(C)** Forest plot demonstrating the results of binary logistic regression analysis. **(D)** Bubble chart of metabolites-specific pathway, calculated by hypergeometric test and topological data analysis (TDA).

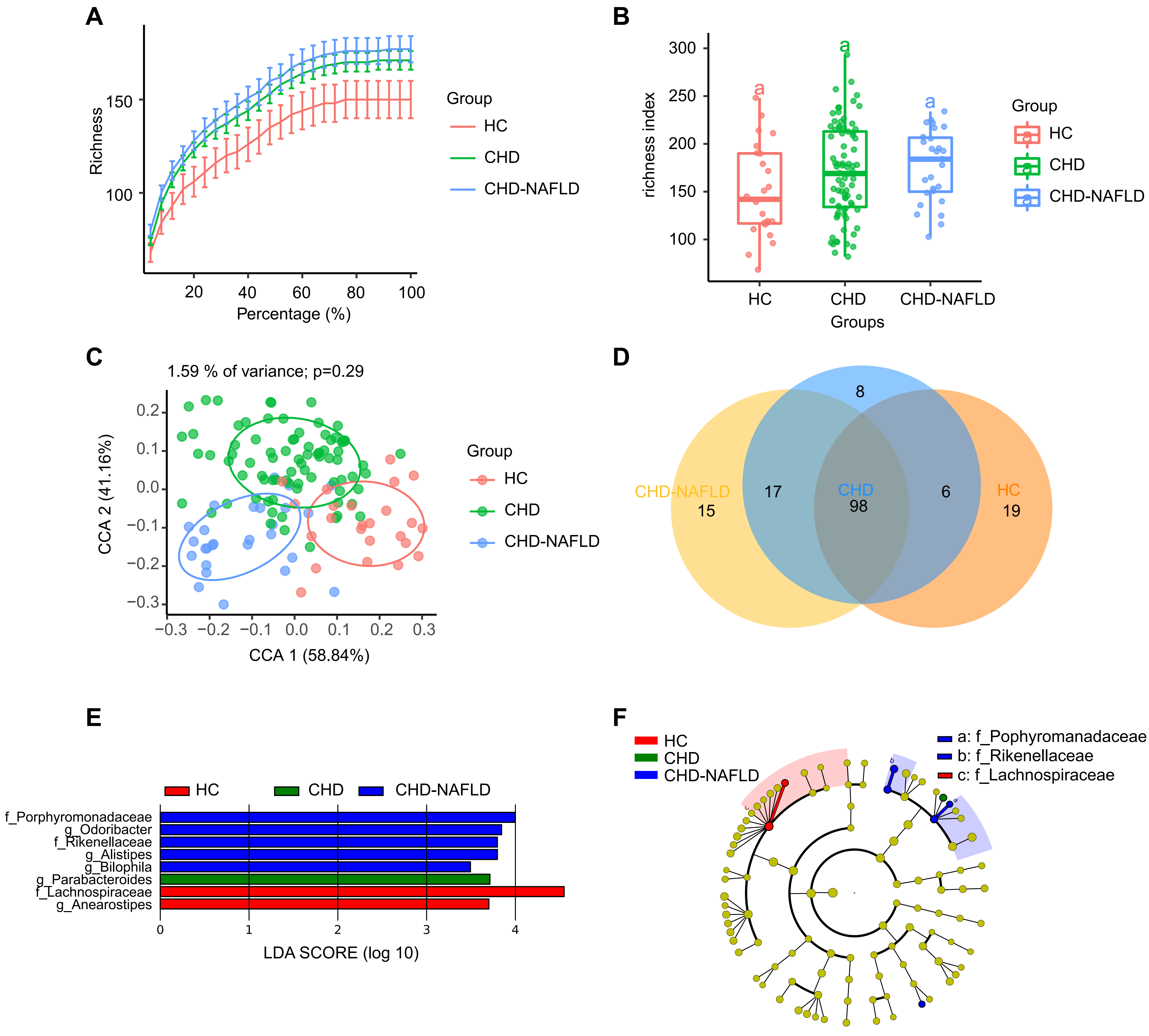

Figure S2. Overview of the gut microbiome in different groups. (A) Dilution curve of HC, CHD, and CHD-NAFLD groups. **(B)** Comparison of richness index between HC, CHD, and CHD-NAFLD groups. **(C)** cPCoA analysis of gut microbiome in three groups. **(D)** A Venn diagram demonstrating the existence of OTUs in each group. **(E-F)** LefSe analysis of gut microbiome in three groups.

Table S1. Key metabolites found in CHD-NAFLD patients.

Module	Compound ID	Alteration	Formula	Description	*P* value	VIP value
module1	PLP4307	enriched	C9H20NO3PS2	FAC	<0.05	1.90685
	PLP4308	enriched	C15H11O6+	Cyanidin cation	<0.05	1.89866
	PLP4309	enriched	C5H4FN3O2	favipiravir	<0.05	1.82169
	PLP558	enriched	C5H15N3O8P2	IMIDO DIPHOSPHATE	<0.05	1.63563
	PLN750	enriched	C15H16Cl3N3O2	Prochloraz	<0.05	1.97355
	PLN2846	enriched	C17H11NO7	Aristolochic acid	<0.05	2.02874
	PLP4598	enriched	C8H5NO	4-Formylbenzonitrile	<0.05	1.59586
	PLP4310	enriched	C14H16BrNO2	brofaromine	<0.05	1.68333
	PLP4595	enriched	C8H9FN2O2S	4-Amidinophenylmethanesulfonyl fluoride	<0.05	1.53841
	PLP4605	enriched	C5H6ClN3	2-Amino-4-chloro-6-methylpyrimidine	<0.05	1.32243
module2	PLP2429	depleted	C21H22FN3O5	(5R)-N-[(2S,3S)-2-(Fluoromethyl)-2-hydroxy-5-oxotetrahydro-3-furanyl]-5-isopropyl-3-(8-isoquinolinyl)-4,5-dihydro-1,2-oxazole-5-carboxamide	<0.05	1.61788
module3	PLP5475	enriched	C6H13NO2	L-Leucine	<0.05	1.93222
	PLP5476	enriched	C5H13NO	5-Amino-1-pentanol	<0.05	2.08768
	PLP5291	enriched	C6H15NO3	Triethanolamine	<0.05	1.67366
	PLP5596	enriched	C4H11NO	Isobutanolamine	<0.05	2.02255
	PLP460	enriched	C23H29NO8	Phenyltoloxamine citrate	<0.05	1.81791
module4	PLP3309	enriched	C15H18N+	Benzyldimethylphenylammonium cation	<0.05	1.56718
	PLP6644	enriched	C18H24N4O	Granisetron	<0.05	1.50371
module5	LPP1260	enriched	C55H90O	all-trans-undecaprenol	<0.05	2.83169
module6	PLP1414	depleted	C16H12N2O2	[5-(9H-beta-Carbolin-1-yl)-2-furyl]methanol	<0.05	1.67658
	PLP5754	depleted	C20H27ClO2	4-Chloromethandienone	<0.05	1.69629
module7	PLP4415	enriched	AlH3O3	Aluminium hydroxide	<0.05	1.34436
module8	PLP5191	depleted	C12H19N2+	Dimethylphenylpiperazinium	<0.05	1.74039
	PLP6057	depleted	C20H40O2	Stearic acid ethyl ester	<0.05	1.82947
	PLP2324	depleted	C13H24O	(Z)-4-Dodecenal	<0.05	1.52775
module9	LPP100	depleted	C9H15NO	9-Methyl-9-azabicyclo[3.3.1]nonan-3-one	<0.05	1.973
	LPP1462	depleted	C4H3NO2	Maleimide	<0.05	2.17097
	PLP3002	enriched	C20H27N5O3	Bamifylline	<0.05	1.39197
module10	PLP209	depleted	C19H34O	2-Pentadecylfuran	<0.05	1.66236
	PLP214	depleted	C16H28O	(10E,12Z)-10,12-Hexadecadienal	<0.05	1.74863
	PLP3242	depleted	C20H38	3-Eicosyne	<0.05	1.45389
	LPN335	depleted	C24H27N8O11PS4	P9VXV1408Y	<0.05	1.60401
module11	PLN2990	depleted	Fe[59]O4S	(~59~Fe)Iron(2+) sulfate	<0.05	2.03041

module12	PLP2633	depleted	C17H15NO6	4',6'-Dimethoxy-2'-hydroxy-3-nitrochalcone	<0.05	1.41822
	PLP2989	depleted	C14H30N4O2	N1,N12-Diacetylspermine	<0.05	1.53884
module13	PLP4269	depleted	C9H14N2O7	(5S,6R)-2'-Deoxyuridine glycol	<0.05	1.74402
	PLP4353	depleted	C15H12N2O3	Disperse Red 11	<0.05	1.53339
	PLP2298	depleted	C10H19NO4	L-Valine, N-[(1,1-dimethylethoxy)carbonyl]-	<0.05	1.93026
	PLP2616	depleted	C19H20N2O2S	2-(1,3-Benzoxazol-2-ylthio)-N-(4-sec-butylphenyl)acetamide	<0.05	1.76982
	PLP6629	depleted	C8H9NO2	N-Phenylglycine	<0.05	1.36533
module14	PLN1465	depleted	C40H54N8O9	nostocyclopeptide A2	<0.05	1.31248
	LPP38	depleted	C22H45O9P	1-Hexadecanoyl-sn-glycero-3-phospho-(1'-sn-glycerol)	<0.05	2.60911
module15	PLN1029	depleted	C16H23NO3	1'-Hydroxybufuralol	<0.05	1.71196
	PLN1726	depleted	C3H7NO4Se	3-Selenino-L-alanine	<0.05	1.96293
	PLN2145	depleted	C22H28FN4O6P	Toceranib phosphate	<0.05	1.66725
	PLN852	depleted	C19H18O7	3'-Hydroxy-5,6,7,4'-tetramethoxyflavone	<0.05	1.74072
	PLP148	depleted	C5H8ClN5	ATRAZINE-DESISOPROPYL	<0.05	1.66478
	PLP1929	depleted	C7H6Cl2	benzal chloride	<0.05	1.56117
	PLP2190	depleted	C10H20N6O4	Asn-Arg	<0.05	1.63299
	PLP2587	depleted	C10H14N2	Anabasine	<0.05	1.72562
	PLP2613	depleted	C14H18N2O5	Aspartame	<0.05	1.74611
	PLP2614	depleted	C10H19N5O5	Arg-Asp	<0.05	1.60654
	PLP2621	depleted	C8H17NO2S	Homocysteine, butyl ester	<0.05	1.4795
	PLP2649	depleted	C16H30O2	Palmitelaidic acid	<0.05	1.6552
	PLP2677	depleted	C16H25NO3	Moxisylyte	<0.05	1.6682
	PLP2848	depleted	C18H35N3O13	chitotriose	<0.05	1.71363
	PLP335	depleted	C13H21NO3	Isoetharine	<0.05	1.79523
	PLP3536	depleted	C6H4Cl2	1,3-Dichlorobenzene	<0.05	1.55957
	PLP3627	depleted	C15H22N2O	Milnacipran	<0.05	1.5723
	PLP3770	depleted	C28H56O2	Octacosanoic acid	<0.05	1.63726
	PLP4345	depleted	C10H12O5	3,4,5-Trimethoxybenzoic acid	<0.05	1.54459
	PLP5677	depleted	C14H30O4S	Myristyl sulfate	<0.05	1.79382
	PLP6101	depleted	C19H20O5	2,2',4',6'-Tetramethoxychalcone	<0.05	1.65668
	PLP6243	depleted	C18H24O2	Estradiol	<0.05	1.59136
	PLP6830	depleted	C19H17ClN4	Fenbuconazol	<0.05	1.53411
	PLP6837	depleted	C4H9NO	N-Methylpropionamide	<0.05	1.61359
module16	PLN2887	depleted	C8H6O4	Terephthalic acid	<0.05	1.58903
module17	PLP6135	depleted	C6H13NO3S	Cyclohexanesulfamic acid	<0.05	1.448
module18	LPP189	enriched	C43H78NO8P	PC(13:0/22:4(7Z,10Z,13Z,16Z))	<0.05	1.9289
module19	PLP3115	enriched	C20H38O	Phytal	<0.05	1.45454
	PLP6349	depleted	C4H9NO2S	S-Methyl-L-cysteine	<0.05	1.65966

	PLP1028	enriched	C14H8N2Na2O6	Azodisal sodium	<0.05	1.54212
	PLP3938	enriched	C6H10O	7-Oxabicyclo[2.2.1]heptane	<0.05	1.89002
module20	PLP6353	enriched	C12H22O11	3-O-(.beta.-D-Galactopyranosyl)-D-glucopyranose	<0.05	1.43999
	PLP6398	enriched	C15H18N4O4	Asn-Trp	<0.05	1.3652
	PLP859	enriched	C21H34NO3+	Oxyphenonium	<0.05	1.44783
	PLP2120	enriched	C37H44N4O6	N1,N5,N10-Tris-trans-p-coumaroylspermine	<0.05	1.46352
	PLP3179	enriched	C25H44O5	Unoprostone isopropyl ester	<0.05	1.70693
	PLP5678	enriched	C6H9NOS	5-(2-Hydroxyethyl)-4-methylthiazole	<0.05	1.44087
	PLN814	enriched	C15H23NO3	Oxprenolol	<0.05	1.66474
	PLP2590	enriched	C19H25NO2	Nylidrin	<0.05	1.90397
module21	PLP2487	enriched	C21H26O2	Cannabinol	<0.05	1.601
module22	PLP3195	depleted	C18H26O4	Compactin diol lactone	<0.05	1.4944
	PLP5058	depleted	C6H12O4	Mevalonic acid	<0.05	1.52244
module23	PLP2066	depleted	C18H32O3	9(R)-HODE	<0.05	1.4368
module24	PLP2727	depleted	NNaO3	Sodium nitrate	<0.05	1.43229
module25	PLP3664	depleted	C37H40N2O6	(1alpha,1'alpha)-6',7,12'-Trimethoxy-2,2'-dimethyloxyacanthan-6-ol	<0.05	1.59871

Supplementary methods

Definition of NAFLD

Nonalcoholic fatty liver disease (NAFLD): We referred to the latest guidelines established by the American Association for the Study of Liver Diseases (AASLD) [1] and the diagnosis of NAFLD is established when the following 4 criteria are met: (1) fatty change of the liver is observed by imaging or histologically; (2) no marked alcohol drinking habit is present (ethanol intake of < 210 g/wk for men and < 140 g/wk for women); (3) no presence of other factors inducing fatty change of the liver; and (4) no concomitant factors causing chronic liver disease are present. B-ultrasound is the preferred method for imaging diagnosis of NAFLD[2]. Liver biopsy is limited by cost, sampling error, and procedure-related morbidity and mortality, and the guidelines do not recommended biopsy except for the following patients: (1) NAFLD patients who are at increased risk to have steatohepatitis and advanced fibrosis; (2) patients with suspected NAFLD in whom competing etiologies for hepatic steatosis and co-existing chronic liver diseases cannot be excluded without a liver biopsy[1]. No such patients were included in this study. Therefore, this study mainly used B-ultrasound for imaging diagnosis of NAFLD.

Statistical analysis of baseline characteristics

The Shapiro-Wilk test was employed to determine the normality of continuous data. Continuous normally distributed data are presented as the mean ± standard deviation (SD). Continuous nonnormally distributed data are presented as the median with interquartile range (IQR). Categorical variables are presented as counts and percentages. For difference comparison of clinical characteristics among the three groups, one-way analysis of variance (ANOVA) was employed in cases of continuous normally distributed data. Bonferroni test was applied for post hoc comparisons in cases of equal variance, and Tamhane test was applied in cases of unequal variance. Kruskal-Wallis H-test was applied for continuous data that were not normally distributed among three groups, and Mann-Whitney U test was applied for this kind of data between two groups. Categorical variables were compared by the $\chi 2$ test or Fisher's exact test (in case of at least one expectation count < 5). The above analyses were performed using SPSS Statistics software, version 24.0 (SPSS Inc., Chicago, IL, USA) and a $P < 0.05$ was considered statistically significant.

References:

1. Chalasani N, Younossi Z, Lavine JE, Diehl AM, Brunt EM, Cusi K et al. The diagnosis and management of non-alcoholic fatty liver disease: practice guideline by the American Gastroenterological Association, American Association for the Study of Liver Diseases, and American College of Gastroenterology. Gastroenterology. 2012; 142:1592-609.
2. Byrne CD, Targher G. NAFLD: a multisystem disease. J Hepatol. 2015; 62:S47-64.
